# Miltefosine Lipid Nanocapsules for Single Dose Oral Treatment of Schistosomiasis Mansoni: A Preclinical Study

**DOI:** 10.1371/journal.pone.0141788

**Published:** 2015-11-17

**Authors:** Maha M. Eissa, Riham M. El-Moslemany, Alyaa A. Ramadan, Eglal I. Amer, Mervat Z. El-Azzouni, Labiba K. El-Khordagui

**Affiliations:** 1 Department of Medical Parasitology, Faculty of Medicine, Alexandria University, Alexandria, Egypt; 2 Department of Pharmaceutics, Faculty of Pharmacy, Alexandria University, Alexandria, Egypt; Universidade Federal do Rio de Janeiro, BRAZIL

## Abstract

Miltefosine (MFS) is an alkylphosphocholine used for the local treatment of cutaneous metastases of breast cancer and oral therapy of visceral leishmaniasis. Recently, the drug was reported in *in vitro* and preclinical studies to exert significant activity against different developmental stages of schistosomiasis mansoni, a widespread chronic neglected tropical disease (NTD). This justified MFS repurposing as a potential antischistosomal drug. However, five consecutive daily 20 mg/kg doses were needed for the treatment of schistosomiasis mansoni in mice. The present study aims at enhancing MFS efficacy to allow for a single 20mg/kg oral dose therapy using a nanotechnological approach based on lipid nanocapsules (LNCs) as oral nanovectors. MFS was incorporated in LNCs both as membrane-active structural alkylphospholipid component and active antischistosomal agent. MFS-LNC formulations showed high entrapment efficiency (EE%), good colloidal properties, sustained release pattern and physical stability. Further, LNCs generally decreased MFS-induced erythrocyte hemolytic activity used as surrogate indicator of membrane activity. While MFS-free LNCs exerted no antischistosomal effect, statistically significant enhancement was observed with all MFS-LNC formulations. A maximum effect was achieved with MFS-LNCs incorporating CTAB as positive charge imparting agent or oleic acid as membrane permeabilizer. Reduction of worm load, ameliorated liver pathology and extensive damage of the worm tegument provided evidence for formulation-related efficacy enhancement. Non-compartmental analysis of pharmacokinetic data obtained in rats indicated independence of antischistosomal activity on systemic drug exposure, suggesting possible gut uptake of the stable LNCs and targeting of the fluke tegument which was verified by SEM. The study findings put forward MFS-LNCs as unique oral nanovectors combining the bioactivity of MFS and biopharmaceutical advantages of LNCs, allowing targeting via the oral route. From a clinical point of view, data suggest MFS-LNCs as a potential single dose oral nanomedicine for enhanced therapy of schistosomiasis mansoni and possibly other diseases.

## Introduction

Neglected tropical diseases (NTDs) afflict more than one billion of the lowest income people in tropical and subtropical regions presenting an enormous global health and economic burden[1]. To overcome slow commercial drug discovery attributed mainly to lack of economic incentives, approaches based on drug repurposing [2] and drug delivery technologies [3] are crucial to bring in effective therapeutics such as amphotericin B [4], phosphodiesterase inhibitors [2]and anticancer drugs [5] to fight NTDs.

A drug of particular interest as repurposing candidate is miltefosine (MFS), a membrane active alkylphosphocholine with a broad pharmacological spectrum [6]. MFS was initially developed as antineoplastic agent but is currently used for the local treatment of cutaneous metastases of breast cancer. Additionally, it has been repurposed with approval in India and more recently, the USA as the first and only oral drug for visceral leishmaniasis. In two earlier studies[7, 8], we demonstrated significant activity of MFS against different developmental stages of *Schistosoma spp*., the causative agent of schistosomiasis, one of the most prevalent chronic NTDs [1]. Unlike praziquantel (PZQ), the mainstay for schistosomiasis control administered in a single oral dose, MFS had to be administered to infected mice in five successive 20mg/kg/day oral doses. This limits its potential clinical applicability as a PZQ alternative for mass chemotherapy. However, overcoming the considerable limitations of PZQ such as poor solubility in water and absorption through the gastrointestinal tract (GIT), failure to prevent re-infection, reduced activity against juvenile worms and development of resistance [9, 10]justify research efforts for novel therapeutic and preventive approaches.

Nanotechnology-based strategies offer great promise in this respect [11]. The schistosomal tegument, as a site of vital and immunological functions for the host response and parasite survival[12],may present a therapeutic target for drugs having antischistosomal activity. This can be supported by the enhanced tegument damage, induced by PZQ nanometric delivery systems via increasing drug concentration and/or activity [13, 14].

MFS is a drug with relatively high bioavailability and long elimination half-life in humans and mice[6, 15]. Although the mechanism of MFS antischistosomal action is not well understood yet, substantial damage of schistosomes by MFS might involve its amphiphilic phospholipid nature and polar quaternary nitrogen[8, 16]. Thus, enhancing interaction with the tegument lipid bilayer membrane using nanotechnological strategies could improve antischistosomal efficacy of MFS. Lipid nanocapsules (LNCs), relatively new nanovectors with a size range 25–100 nm, were shown to increase the bioavailability and activity of orally administered drugs [17]. They consist of a lipid core surrounded by a relatively rigid shell of lecithin and pegylated surfactant that provides stealth properties. LNCs, prepared by a low-energy solvent-free method, show good dispersion stability and maintain structural integrity in artificial gastrointestinal media [18]. Further, Caco-2 cells studies revealed LNCs internalization mainly via active endocytic processes that likely allow transcytosis and gastrointestinal transport of the encapsulated drug [19].

The objective of the present study was to achieve effective treatment of *Schistosoma mansoni*-infected mice by the administration of a single 20 mg/kg oral dose of MFS lipid nanocapsules using a formulation-based approach. MFS was incorporated both as a membrane active phospholipid component of the LNCs shell structure and as an active antischistosomal agent.

## Methods

### Ethics Statement

All animal studies were conducted at the Animal Experimentation Facility of the Medical Parasitology Department, Faculty of Medicine, Alexandria University, Egypt, in strict accordance with the Faculty of Medicine Guidelines for ethical conduct in use of animals in research. Efforts were made to minimize animal suffering. The study protocols were approved by the Ethics Committee of the Faculty of Medicine, Alexandria University (Protocol approval numbers 0302440 and 0302474 for the antischistosomal activity study in mice and the pharmacokinetic study in rats respectively). In the *in vitro* hemolysis study, a 2 ml fresh blood sample was donated by one of the authors under medical supervision.

### Materials

Miltefosine (MFS, 1-hexadecylphosphocholine) was obtained from Chem-Impex International Inc.(500 Fifth Avenue, Suite 2440, New York, USA). Labrafac lipophile WL 1349 (caprylic-capric acid triglycerides, European Pharmacopeia, IVth, 2002) was a kind gift from Gattefossé S.A. (Saint-Priest, France). Solutol^®^ HS 15 (a mixture of free polyethylene glycol 660 and polyethylene glycol 660 hydroxystearate, European Pharmacopeia, IVth, 2002) was provided by BASF (Ludwigshafen, Germany). Lipoid S100 (a soybean lecithin, 69% of phosphatidylcholine) was donated by Lipoïd GMBH(Ludwigshafen, Germany). Hexadecyltrimethyl ammonium bromide (CTAB), dihexadecyl phosphate (DCP) and oleic acid (OA) were purchased from Sigma Aldrich (USA). Chitosan (CS, Protasan UP) was purchased from Novamatrix, Sandvika, Norway. All other chemicals were of analytical grade.

### Preparation of lipid nanocapsules

Lipid nanocapsules (LNCs) were prepared by the phase inversion method [18]. In brief, Solutol, Labrafac, deionized water (1:1:3) and sodium chloride(0.44% w/v of the final volume) were weighed and mixed under magnetic stirring. The mixture was subjected to three cycles of progressive heating and cooling between 65 and 85°C (45 to 65°C for LNCs formulations containing oleic acid (OA) at a rate of 4°C/minute. An irreversible shock was induced by 4-fold dilution with deionized cold water (0–2°C) added to the oil in water (o/w) emulsion at a temperature 1–3°C from the beginning of the phase inversion zone (PIZ). This was followed by slow magnetic stirring of the LNCS dispersion for 5 min. For loaded formulations, MFS (0.7 mM % of the final dispersion volume) was added just before quenching. The additives, dicetyl phosphate (DCP), hexadecyltrimethyl ammonium bromide(CTAB) and OA were mixed with other ingredients at the beginning of the process. For chitosan(CS)-coated LNCs, a post-insertion technique [20] involving incubation of preformed LNCs at room temperature with CS solutions of variable concentrations (0.5–1% of the final dispersion) for 2 h under low speed stirring was used. Six test LNCs formulations (Table 1) were tested.

**Table 1:** Test LNC formulations, their codes and formulation additives.

LNC formulations	Code	Additive (s)	Additive (% w/v)^a^
**LNC (standard)** ^b^	LNCs	-	-
**LNC-CTAB (cationic)**	LNC-CTAB^+^	CTAB	0.500
		Lipoid S100	75
**LNC-chitosan (cationic)**	LNC-CS^+^	Chitosan	0.50
**LNC-DCP (anionic)**	LNC-DCP^¯^	DCP	0.50
**LNC- oleic acid** ^c^	LNC- OA	Oleic acid	1.00
**LNC-CTAB and oleic acid (cationic)**	LNC-OA-CTAB^+^	CTAB	0.50
		Oleic acid	1.00

^a^ % w/v of the final volume of the preparation

^b^ MFS-LNCs not containing additives

^c^ Standard LNCs and blank LNC-CTAB^+^ and LNC-OA not containing MFS were used as controls in the animal study

### In vitro characterization of lipid nanocapsules

The mean particle size and zeta potential of LNC formulations were measured by photon correlation spectroscopy (PCS Zetasizer^®^Nano ZS Series DTS 1060, Malvern Instruments S.A., Worcestershire, UK) at a fixed angle at 25°C using a 4 mW He–Ne laser at 633 nm. LNCs dispersion was diluted 1:80 in deionized water and measurements were performed in triplicate. The stability of MFS-loaded LNCs regarding particle size, polydispersity index (PdI) and zeta potential was assessed after a 4 month-storage period at 4°C.

The morphology of LNCs was examined by transmission electron microscopy (TEM) using JEOL, JEM-100 CX Electron Microscope, Tokyo, Japan. Before analysis, the selected LNC dispersions were treated with a negative stain (2% w/v uranyl acetate solution) and sprayed onto copper grids. Shots were taken at X 7500 at 80 kV.

Entrapment efficiency (EE%) was calculated by determining the concentration of free MFS (un-entrapped) in the ultrafiltrate after separation of LNCs using an ultrafiltration / centrifugation technique (Sigma 3-30KS, Sigma Laborzentrifugen GmbH, Germany). A 5 ml-sample of LNC dispersion was added to the ultracentrifugal concentrator (Sartorius^™^ Vivaspin6^™^, MWCO 100,000) and centrifuged for 30 min at 3663 x g. A modified spectrophotometric assay reported for quaternary ammonium compounds using bromothymol blue (BTB) [21] was used for MFS determination in the ultrafiltrate. An aliquot of the ultrafiltrate was mixed with hydroxypropyl methylcellulose (HPMC) solution (0.4% w/v), BTB solution in methanol (0.06% w/v) and phosphate buffer saline pH 7.5 in the ratio 4:4:1:41 by volume. The color developed was measured at 616 nm. MFS concentration was calculated using calibration standards and blank LNC filtrates obtained under similar conditions. Linearity was checked within the 1–40 μg/ml range. Measurements were done in triplicate. The concentration of MFS in LNCs was calculated from the difference between the initial drug concentration and the free drug concentration in the ultrafiltrate. The same procedure was used to assess the effect of storage at 4°C for 4 months on the leakage of MFS from LNCs.

The release of MFS was determined at 37°C at 100 rpm in phosphate buffer saline (PBS, pH 7.4). A known volume of selected MFS-LNC dispersions was diluted to 5 ml with PBS to achieve sink conditions. At different time intervals, LNCs were separated and the whole filtrate was used to determine MFS concentration spectrophotometrically as described above. The % MFS released was calculated in triplicate relative to the theoretical initial drug content.

### In vitro hemolytic activity

The erythrocyte hemolytic activity of MFS-LNCs as a surrogate indicator of membrane activity was assessed *in vitro* using a reported method [22]. Briefly, fresh human blood (2 mL) was collected using EDTA as anticoagulant and centrifuged at 2500 rpm for 10min. RBCs were separated, washed three times with PBS pH 7.4 and diluted with PBS to obtain a 1% v/v hematocrit suspension. Aliquots of a stock MFS solution (0.15 mg/100mL) or selected MFS-LNCS were mixed with 2 mL of the hematocrit suspension to provide mixtures containing 25–100 μM of MFS. The mixtures were incubated for 45 min at ambient temperature (~25°C) then centrifuged at 2500 rpm for 10 min. Optical density was measured at 540 nm. Positive and negative controls were prepared by incubating hematocrit with 0.1% w/v Triton X-100 and PBS, respectively under similar conditions. The % hemolysis was calculated as follows:

$$\text{\% Hemolysis= }\frac{\text{As-An}}{\text{As-Ap}}\text{x100}$$

where, As, An and Ap are the absorbance readings of the sample, negative control and positive control, respectively.

### Preclinical study in *Schistosoma mansoni*-infected mice

Mice were housed under specific pathogen-free barrier conditions. The Egyptian strain of *S*. *mansoni*, kept at the Department of Medical Parasitology, Faculty of Medicine, Alexandria University, Egypt was used. It was propagated in *Biomphalaria alexandrina* snails as the intermediate host. Cercariae of *S*. *mansoni* were obtained from the snails infected with miracidia from the feces of mice infected 45 days prior to the study. Forty days later, the snails were subjected to illumination at 28°C to induce cercarial shedding. The numbers and viability of cercariae were determined by light microscopy.

#### Host animals and experimental groups

Male Swiss albino mice 4–5 weeks old and weighing 20–25 g, obtained from the animal house, Medical Parasitology Department, Faculty of Medicine, Alexandria University, Egypt, were used as the definitive host. Mice were infected by the tail immersion method[23] using a cercarial suspension at 100 cercariae per animal. Animals were kept for 42 days under daily care. Mice were then randomly divided into study groups, 6 mice each, corresponding to the study formulations and controls (Table 1). Mice in the treatment groups were administered a single 20 mg/kg oral dose of MFS either as an aqueous solution or LNCs aqueous dispersion by gastric gavage.

#### Assessment of antischistosomal efficacy

Mice of all groups were sacrificed by cervical dislocation after a heparin injection seven days post-treatment (on 49 day p.i.). The antischistosomal activity of formulations of the treatment groups compared to the appropriate control groups was assessed by determination of the % reduction in total worm burden and size of liver granulomas, histopathological changes in liver parenchyma and examination of the morphology of recovered worms by TEM and SEM(JFC-1100E (JEOL, Japan).

The adult worm burden was estimated by recovering schistosomes from the mice hepatic portal system by perfusion with citrated saline[23]. The total worm counts in treated and control mice were used to determine the % reduction in worm burden.

For histolopathological examination, specimens of the liver of mice of all study groups were fixed in 10% neutral buffered formalin. Then, 5 μm-thick histological sections were stained with haematoxylin and eosin. Pathological changes in the hepatic parenchyma were noted and the mean diameters of granulomas (μm)determined. Only granulomas containing one clearly identifiable, central-only egg were selected [24].

Morphological examination of adult schistosomes recovered from mice of the infected untreated control group and the treatment group showing the highest % reduction in worm burden was performed by SEM and TEM after fixation in cold 1.5% glutaraldehyde[25].

### Pharmacokinetic study

The study was conducted on four selected MFS-LNC formulations: standard MFS-LNCs and three modified formulations, namely MFS-LNCs-OA, MFS-LNCs-CTAB^+^ and MFS-LNCs-OA-CTAB^+^ (Table 1) using MFS solution as control. Male Sprague-Dawley rats (weight 270–290 g, obtained from the animal house of Pharos University, Alexandria, Egypt) were fasted overnight prior to the experiment. Animals were divided into five groups (n = 5). Rats in the treatment groups were administered a single 10 mg/kg oral dose of MFS either as an aqueous solution or MFS-LNCs dispersion by gastric gavage. This dose was equivalent to the 20mg/kg MFS dose administered to mice in the preclinical study after correction for rats [26]. Following administration, blood samples were collected via the orbital plexus under anesthesia at time intervals of 0.5, 1, 2, 4, 7, 10, 24, 48, 72 and 216 h in Eppendorf tubes containing EDTA. Blood samples were then centrifuged immediately at 4000 rpm for 10 min. Plasma samples were frozen and maintained at -80°C pending analysis.

#### Quantification of MFS in plasma samples

MFS in plasma samples was analyzed using a reported sensitive and selective LC-MS/MS method for the quantification of MFS in plasma samples with slight modification [27]. An aliquot (100 μL) of rat plasma sample was vortex-mixed with 900 μl methanol (HPLC grade, Sigma Aldrich) for 1 min then centrifuged at 5000 rpm for 10 min. The method was validated for linearity, intra-day and inter-day precision and limits of detection and quantification. Ekspert TMultraC 100, Eksigent system with autosampler and column compartment was used. Separation was carried out on ZORBAX Eclipse Plus C18 column (4.6 x 150 mm, 5 μm). An isocratic eluent consisting of 10mM ammonia in water (mobile phase A) and ammonia in methanol (mobile phase B) in a ratio of 5:95 v/v was used. The injection volume was 10 μL and the flow-rate was adjusted to 1 mL/min.

Mass spectrometric detection was performed on SCIEX Triple Quad^™^ 5500 Mass Spectrometer. The mass transition of m/z 408 to 124.8 was optimized for MFS with a dwell-time of 1500 ms. Curtain gas (nitrogen grade 5.0) was set at 20 psi and the collision gas (nitrogen grade 5.0) at 8 psi. The ion spray voltage was set at 5500V, while the source temperature was 600°C. Quantification was achieved using a calibration curve for peak area ratios of MFS in spiked plasma obtained under the same conditions.

### Pharmacokinetic data analysis

The plasma concentrations versus time data were analyzed based on a non-compartmental pharmacokinetic model using Excel pharmacokinetic solver add-in [28]. The main pharmacokinetic parameters for all groups were calculated. Results were expressed as mean ± standard deviation (SD) (n = 5).

### Statistical analysis

Data of the preclinical study in mice were analyzed using IBM *SPSS software package version 20*.*0*. Quantitative data were described using the parameters: range (minimum and maximum), mean, standard deviation and median. Comparison between different groups was analyzed using F-test (ANOVA) and Post Hoc test (LSD) for pair-wise comparison. Significance test results were quoted as two-tailed probabilities. In the pharmacokinetic study, data were analyzed using a paired t-test. In both studies, p values ≤0.05indicated significance.

## Results

### Characterization of lipid nanocapsules

Table 2 shows the physicochemical properties of LNC formulations. Standard LNCs not containing additives were generally uniform with a mean diameter of58.7 nm. Inclusion of additives modified surface charge and had a limited effect on particle size (55.0–58.9 nm) and PdI except CTAB which significantly (p<0.05) reduced particle size. Cationic additives increased zeta potential, the effect of CTAB was greater than that of chitosan (CS). MFS loading did not noticeably affect LNCs properties.

**Table 2:** Properties of blank LNCs and MFS-LNC formulations (n = 3).

Formulation code	Blank LNCs^a^			MFS-LNCs		
	Mean size nm± SD	PdI^b^	Zeta pot. mV ± SD	Mean size nm± SD	PdI^b^	Zeta pot. mV ± SD
**LNCs** ^c^	58.7±2.03	0.058	-5.9±1.16	62.1±0.92	0.095	-0.9±3.06
**LNC-CTAB+**	48.7±0.84	0.280	30.4±6.14	44.0±0.83	0.166	36.5±2.80
**LNC-CS+**	58.9±0.78	0.081	15.5±3.11	64.5±0.76	0.099	14.3±4.52
**LNC-DCP¯**	55.0±0.39	0.041	-25.9±3.89	55.6±0.42	0.051	-22.7±5.18
**LNC-OA**	56.5±0.12	0.022	-9.1±0.36	53.3±2.61	0.068	-7.1±1.22
**LNC-OA-CTAB+**	40.5±0.92	0.062	32.8±3.44	39.08±1.61	0.082	38.0±7.07

^a^ Not containing MFS

^b^ Polydispersity index

^c^ Not containing additives

Storage of LNCs dispersions for 4 months at 4°C did not significantly change colloidal properties (results not shown). With the exception of MFS-LNC-DCP¯ and MFS-LNC-CTAB^+^ which showed a slightly but significantly (p<0.05) larger mean diameter (68.42±0.28 and 53.8±1.10 nm, respectively), LNCs remained monodisperse with PdI< 0.3. Formulations incorporating oleic acid (MFS-LNC-OA and MFS-LNC-OA-CTAB^+^) showed no significant increase in particle size (56.21±0.3 and 39.64±0.19 nm respectively). Zeta potential did not significantly change upon storage.

**Fig 1:**
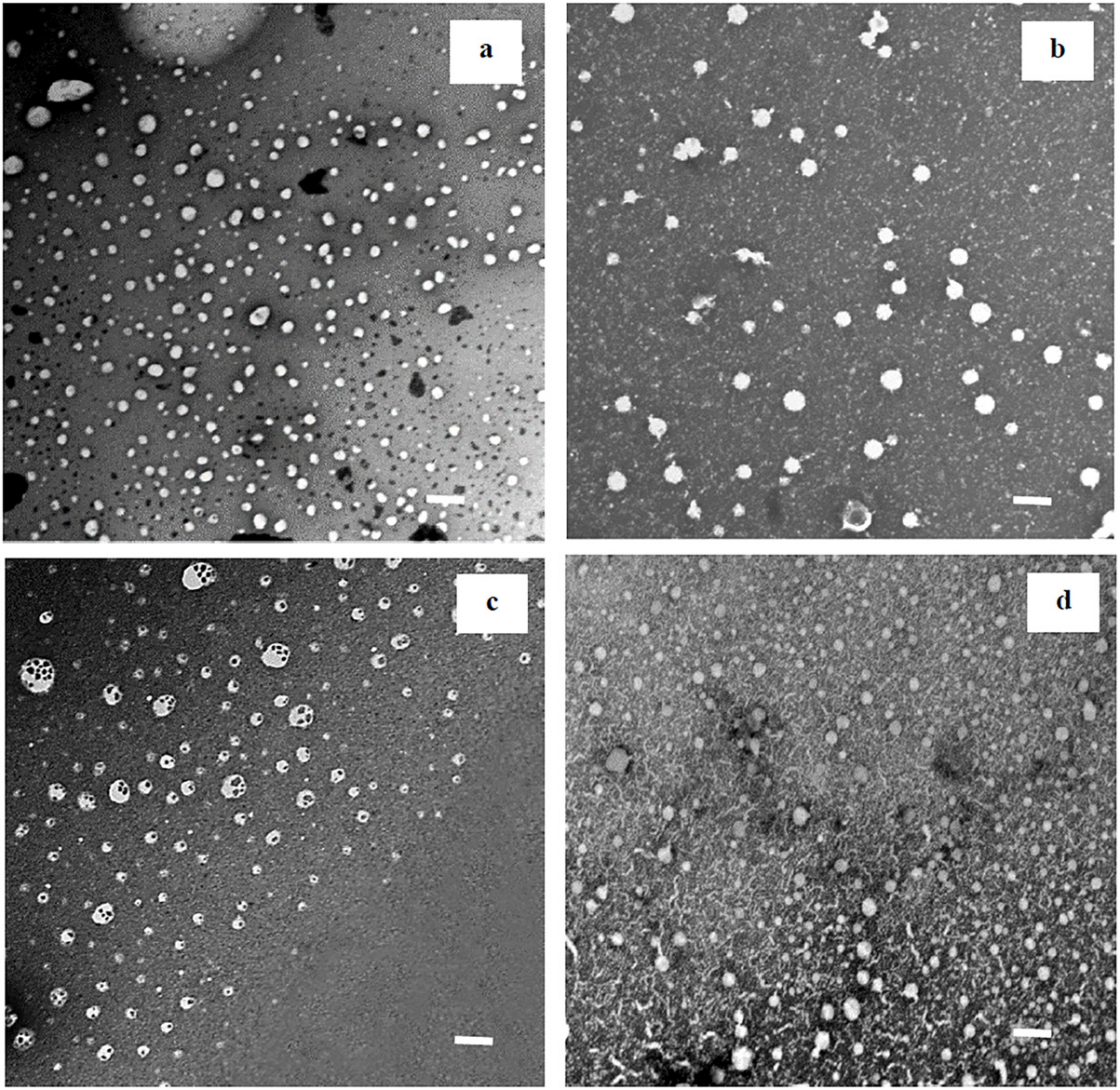
TEM images of miltefosine lipid nanocapsules (MFS-LNCs). .(a) Plain MFS-LNCs, (b) MFS-LNC-CTAB^+^, (c) MFS-LNC-OA and (d) MFS-LNC-OA-CTAB^+^ at X 7,500 magnification. The scale bar represents 100nm.

Fig 1a–1d shows TEM images of standard MFS-LNCs, MFS-LNC-CTAB^+^, MFS-LNC-OA and MFS-LNC-OA-CTAB^+^, respectively. With the exception of MFS-LNC-OA, LNCs were almost spherical, homogenously distributed and not aggregated. MFS-LNC-OA (Fig 1c) showed vesicle-like composite structures with different morphological characteristics.

Entrapment efficiency values (EE%) exceeded 97.5% in all LNC formulations with an average drug payload of ~25.6 mg/g. Reassessment of MFS EE% following 4 month-storage at 4°C indicated no significant (p>0.05) leakage from MFS-LNC-OA and MFS-LNC-OA-CTAB^+^ (EE % was 96.5±1.31and 95.6±0.95 respectively). However, a limited but significant (p<0.05) reduction in EE% was observed for standard MFS-LNC, MFS-LNC-CTAB^+^ and MFS-LNC-DCP^-^ to 93.4±2.05, 94.1±0.98 and 93.8±1.25%, respectively.

**Fig 2:**
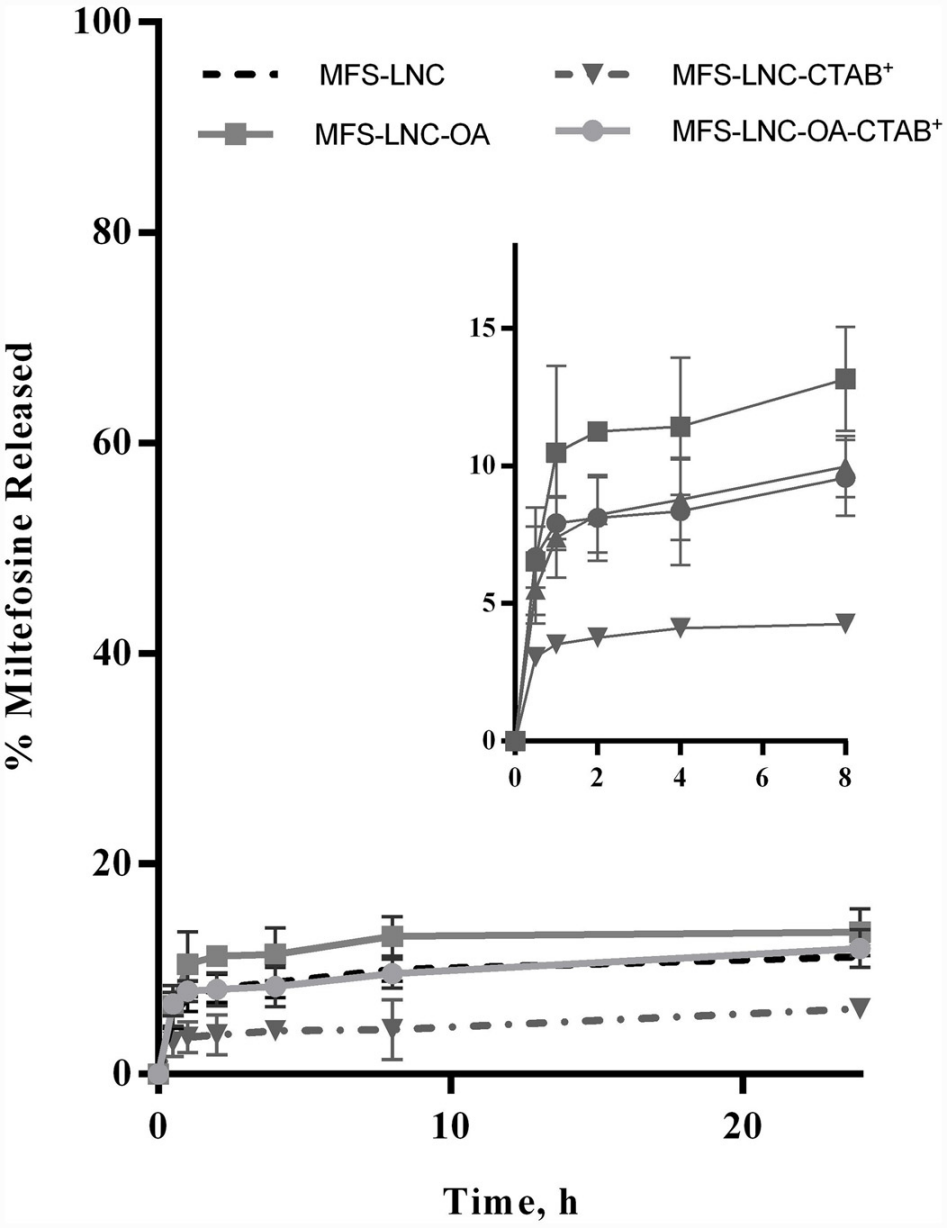
Miltefosine release profiles. Miltefosine release from selected lipid nanocapsule formulations in PBS pH 7.4 at 37°C over a 24 h-study period. Data represent mean ± SD (n = 3). The inset is an enlargement of the release profiles for the first 8h of the release study.

*In vitro* MFS release in PBS pH 7.4 at 37°C under sink conditions (Fig 2) for 24h showed sustained release profiles with a relatively small burst effect. Compared to standard MFS-LNC, the % MFS release rate was reduced by the cationic surfactant CTAB (MFS-LNC-CTAB^+^) and increased by oleic acid (MFS-LNC-OA) to a limited extent, nonetheless, differences at 2, 4, 8 and 24 h were statistically significant (p<0.05).

### *In vitro* hemolytic activity as indicator of membrane activity

**Fig 3:**
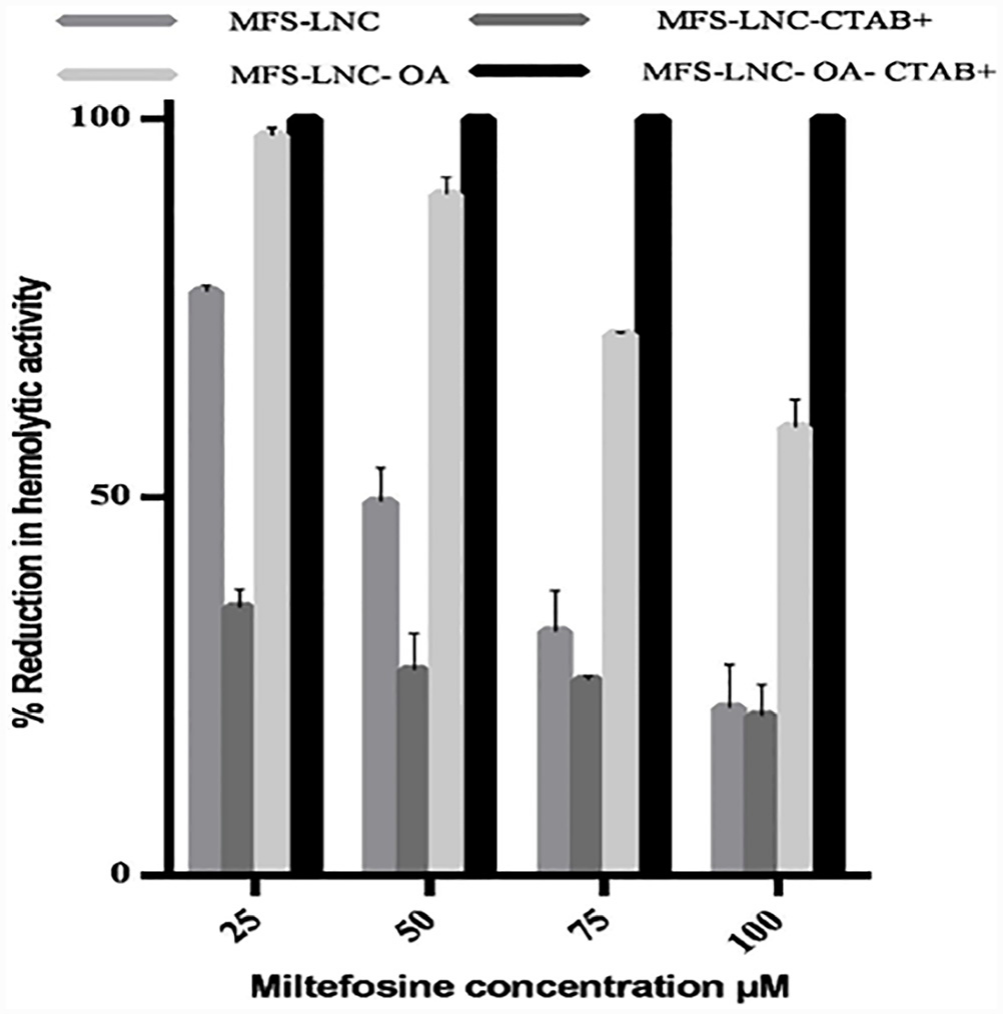
*In vitro* hemolytic activity of miltefosine lipid nanocapsules. Reduction of the *in vitro* hemolytic activity of MFS at different concentration levels by selected LNC formulations. Data represent mean ± SD (n = 3).

MFS solution in the 25–100 μM range induced 100% erythrocytes hemolysis upon 45 min incubation at ambient temperature (~25°C). LNCs (standard MFS-LNCs, MFS-LNC-CTAB^+^, MFS-LNC-OA, and MFS-LNC-OA-CTAB^+^) reduced MFS hemolytic activity, the effect being dependent on both MFS concentration and LNCs composition (Fig 3). Hemolysis reduction was significant (p<0.05) for all systems except MFS-LNC-CTAB^+^ at 100 μM MFS. LNCs incorporating both CTAB and oleic acid prevented hemolysis at all MFS concentrations. CTAB control induced minimal hemolysis.

**Fig 4:**
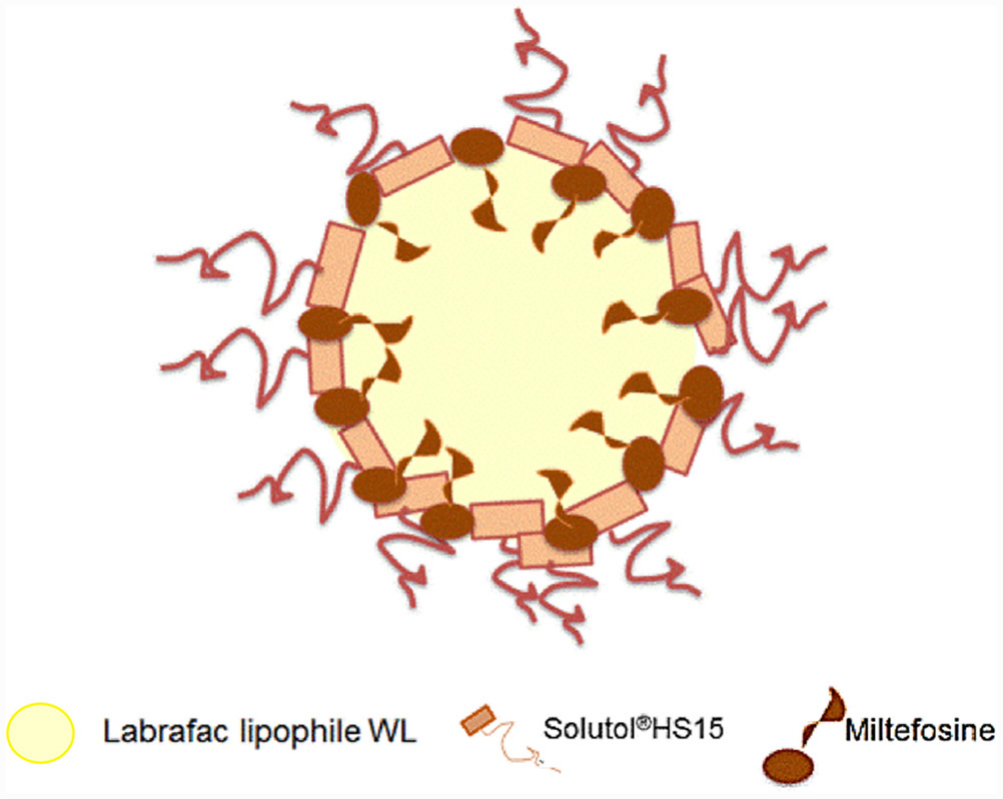
Hypothetical diagram of lipid nanocapsules incorporating miltefosine.

Based on the characterization data obtained, a diagram for MFS-LNCs was hypothesized (Fig 4).

### Antischistosomal activity in *Schistosoma mansoni*-infected mice

Data for worm burden and their statistical analysis are shown in Table 3. Treatment of *S*.*mansoni*-infected mice with either drug-free LNCs (data not shown) or MFS solution (MFS-sol) did not significantly change the mean worm burden compared to un-treated mice.However, administration of the six MFS-LNC formulations reduced the mean worm burden to different extents (42.31 to 88.46%). While cationic MFS-LNCs (MFS-LNC-CTAB^+^) and those containing oleic acid (MFS-LNC-OA) induced the greatest reduction, the least reduction was induced by the negatively charged MFS-LNC-DCP^-^. In all cases, the mean worm burden for MFS-LNC formulations was significantly lower compared to that for MFS-sol or the untreated control groups. The difference between% reduction among MFS-LNCs treatment groups was significant while the difference between MFS-LNC-CTAB^+^ and MFS-LNC-OA did not reach significance.

**Table 3:** Effect of miltefosine lipid nanocapsule formulations (MFS-LNCs) on *S*. *mansoni* worm burden as compared with control groups(n = 6). F* = 78.369 (p<0.001).

			Animal group / Formulation code					
Worm load	Infected untreated Control	MFS-sol Control	Group I	Group II	Group III	Group IV	Group V	Group VI
			MFS-LNC	MFS-LNC- CTAB^+^	MFS-LNC- CS	MFS-LNC- DCP^¯^	MFS-LNC- -OA	MFS-LNC- OA-CTAB ^+^
**Min.**	37.0	34.0	7.0	4.0	6.0	15.0	2.0	15.0
**Max.**	41.0	37.0	19.0	8.0	14.0	30.0	6.0	22.0
**Mean**	39.0	35.83	12.67^a^ ^b^	5.17^a^ ^b^ ^c^	10.0^a^ ^b^ ^d^	22.50^a^ ^b^ ^c^ ^d^ ^e^	4.50^a^ ^b^ ^c^ ^e^ ^f^	18.0^a^ ^b^ ^c^ ^d^ ^e^ ^f^ ^g^
**SD**	1.41	1.17	5.01	1.83	3.41	6.28	1.64	2.61
**Median**	39.0	36.0	12.0	4.0	10.0	22.50	5.0	17.50
** % R**_**1**_		8.13	67.51	86.74	74.36	42.31	88.46	53.85
** % R**_**2**_			64.64	85.57	72.09	37.20	87.44	49.76

* F test (ANOVA). % R_1_: % reduction in each of the study groups relative to infected control. %R_2_:% reduction in each of the study groups relative to miltefosine solution control

^a:^ significant with infected untreated control

^b:^ significant with MFS solution control

^c:^ significant with group I

^d:^ significant with group II

^e:^ significant with group III

^f:^ significant with group IV

^g:^ significant with group V

Regarding granuloma size, MFS-LNC formulations brought about a reduction in granulomas diameter (μm) ranging from 17.1 to 31.4% (Table 4). The largest reduction was induced by MFS-LNC-CTAB^+^(31.36%) and MFS-LNC-OA (32.99%) although the difference between both formulations was not statistically significant. In all cases, the difference in granuloma diameters was statistically significant when MFS-sol or the infected untreated group was used for comparison. None of the corresponding blank formulations had an effect on granuloma size (data not shown).

**Table 4:** Effect of miltefosine lipid nanocapsule formulations (MFS-LNCs) on hepatic granulomas size in μm as compared with control groups. F* = 110.924 (p<0.001).

			Animal group / Formulation code					
Granuloma size, μm	Infected untreated control	MFS-sol control	Group I	Group II	Group III	Group IV	Group V	Group VI
			MFS-LNC	MFS-LNC -CTAB	MFS-LNC -CS	MFS-LNC -DCP^-^	MFS-LNC- -OA	MFS-LNC -OA-CTAB
**Min.**	285.0	280.0	232.0	206.0	214.0	245.0	190.0	235.0
**Max.**	325.0	325.0	240.0	215.0	222.0	260.0	220.0	245.0
**Mean**	305.94	299.64	236.0^a^ ^b^	210.0^a^ ^b^ ^c^	218.17^a^ ^b^ ^c^	253.50^a^ ^b^ ^c^ ^d^ ^e^	205.0^a^ ^b^ ^c^ ^e^ ^f^	240.67^a^ ^b^ ^d^ ^e^ ^f^ ^g^
**SD**	13.49	15.84	2.65	3.16	2.71	5.43	11.90	4.41
**Median**	303.75	300.0	236.0	209.50	218.0	254.0	210.0	241.50
**% R**_**1**_		2.06	22.86	31.36	28.69	17.14	32.99	21.33
**% R**_**2**_			21.24	29.92	27.19	15.40	31.58	19.68

* F test (ANOVA), % R_1_: % reduction in each of the study groups relative to infected control %R_2_: % reduction in each of the study groups relative to miltefosine solution control

^a:^ significant with infected untreated control

^b:^ significant with MFS solution control

^c:^ significant with group I

^d:^ significant with group II

^e:^ significant with group III

^f:^ significant with group IV

^g:^ significant with group V

**Fig 5:**
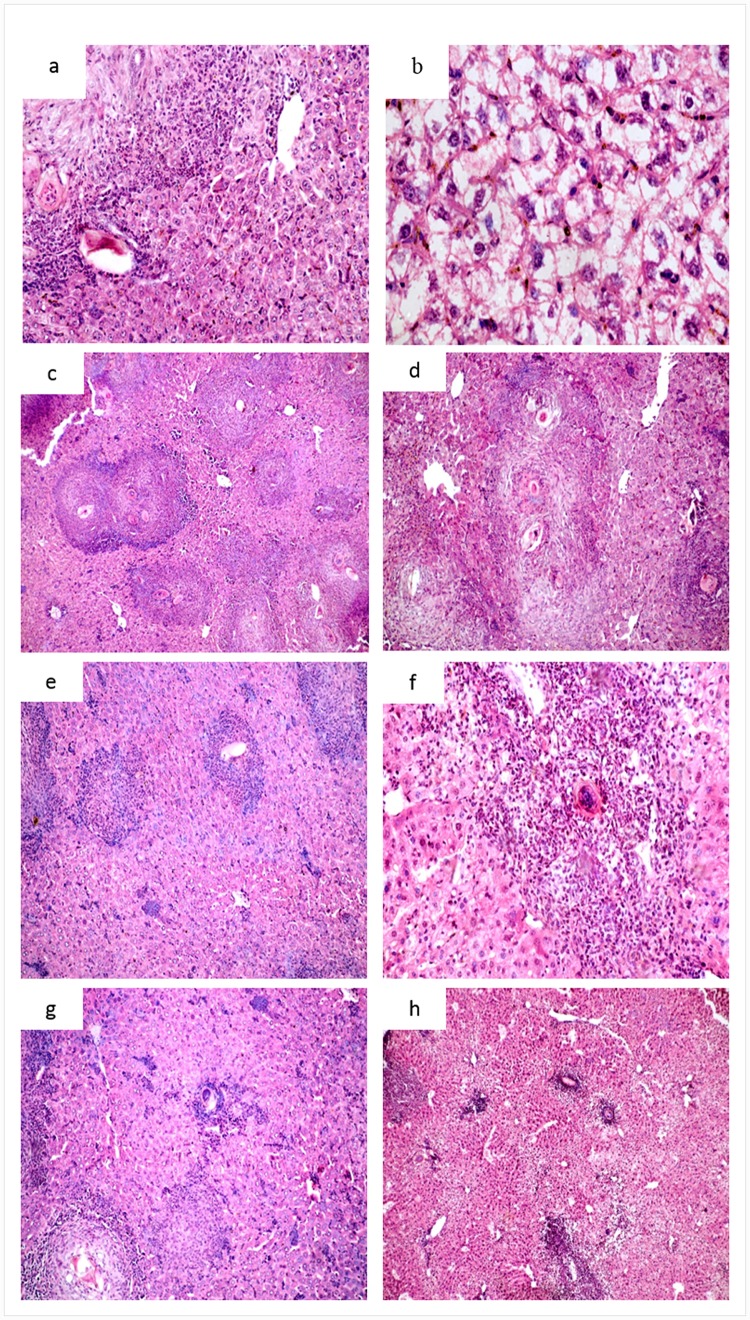
Histopathological study of H&E stained liver sections of different groups of mice infected with *Schistosoma mansoni*. **Infected untreated mice showing:**(a) preserved hepatic acinar architecture, intense inflammatory infiltration and Kupffer cell hyperplasia (X200); (b) Marked ballooning and swelling of hepatocytes (X400); (c), (d) and (e) several granulomas consisting of epithelioid, eosinophilic and lymphocytic cells surrounding well developed eggs (X100, X200 and 400 respectively). **Infected, MFS-LNCS-OA-treated mice showing:** (f), (g) and (h) small cellular granulomas with mild degree of cellular swelling and inflammatory infiltration (X100, X200 and X100 respectively).

Histopathological changes in liver sections of infected untreated mice are shown in Fig 5a–5e Preserved hepatic acinar architecture, intense inflammatory infiltration, marked dilatation of blood sinusoids and Kupffer cells hyperplasia are clear in Fig 5a while Fig 5b shows cellular swelling, Further, several cellular granulomas in both portal tracts and parenchymas were observed(Fig 5c, 5d and 5e). They consisted of epithelioid and lymphocytic cells and encircling lateral spined eggs with intact egg shell On the other hand, liver sections from all mice groups treated with MFS-LNC formulations showed smaller cellular granulomas as compared to the control. The smallest granulomas were observed in mice treated with MFS-LNC-OA (Fig 5f, 5g and 5h). Eggs inside the granulomas were degenerated with irregular shell. The internal structures were either ill-defined or absent. The associated parenchymal pathological changes were milder than those of the control.

**Fig 6:**
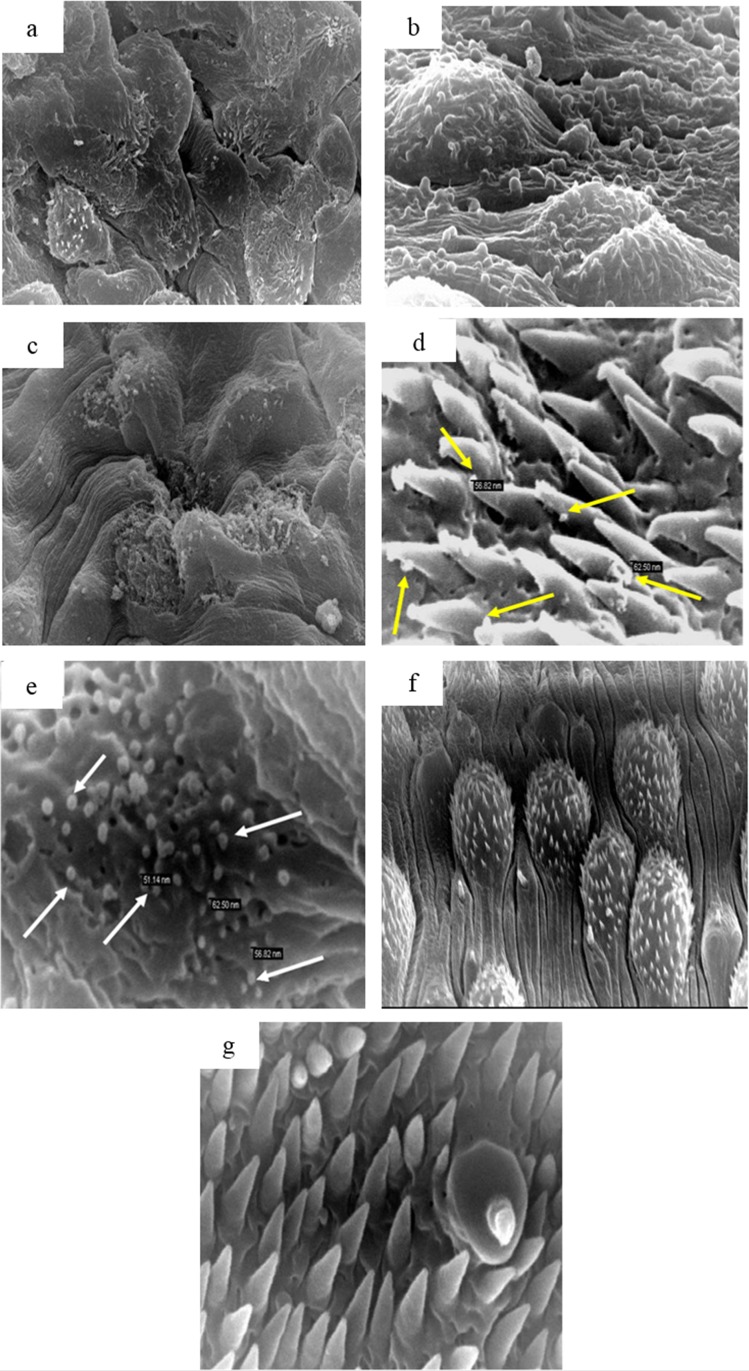
Scanning Electron Microscopy (SEM) of a male *Schistosoma mansoni* worm from a mouse treated with MFS-LNC-OA showing. (a) Marked tegumental irregularity and disfigurement (X 3,500); (b) Tegumental surface blebbing (X 7,500); (c) Edema, flattening and sloughing of the whole tubercles with partial to complete loss of the spines (X 5,000); (d) and (e) nano-objects of similar size to lipid nanocapsules in between spines and on damaged schistosomal surface, respectively (X35,000). SEM of a normal male worm showing: (f) and (g) normal dorsal tegumental surface and papilla (X5,000 and 35,000 respectively)

SEM of male worms recovered from mice treated with MFS-LNC-OA, the test formulation inducing the highest % reduction in worm burden are shown in Fig 6a–6c. Apparent extensive damage to the tegumental surface (Fig 6a) was characterized by surface blebbing (Fig 6b), marked disfigurement and flattening of dorsal tubercles with loss of tubercle spines (Fig 6c). Interestingly, SEM of the fluke tegument obtained at X 35,000 (Fig 6d and 6e)showed nano-objects of the LNCs size between spines (Fig 6d) and on the damaged tegument surface (Fig 6e). SEM of the dorsal tegumental surface of a normal *S*. *mansoni* male worm (Fig 6f and 6g) is shown for comparison.

**Fig 7:**
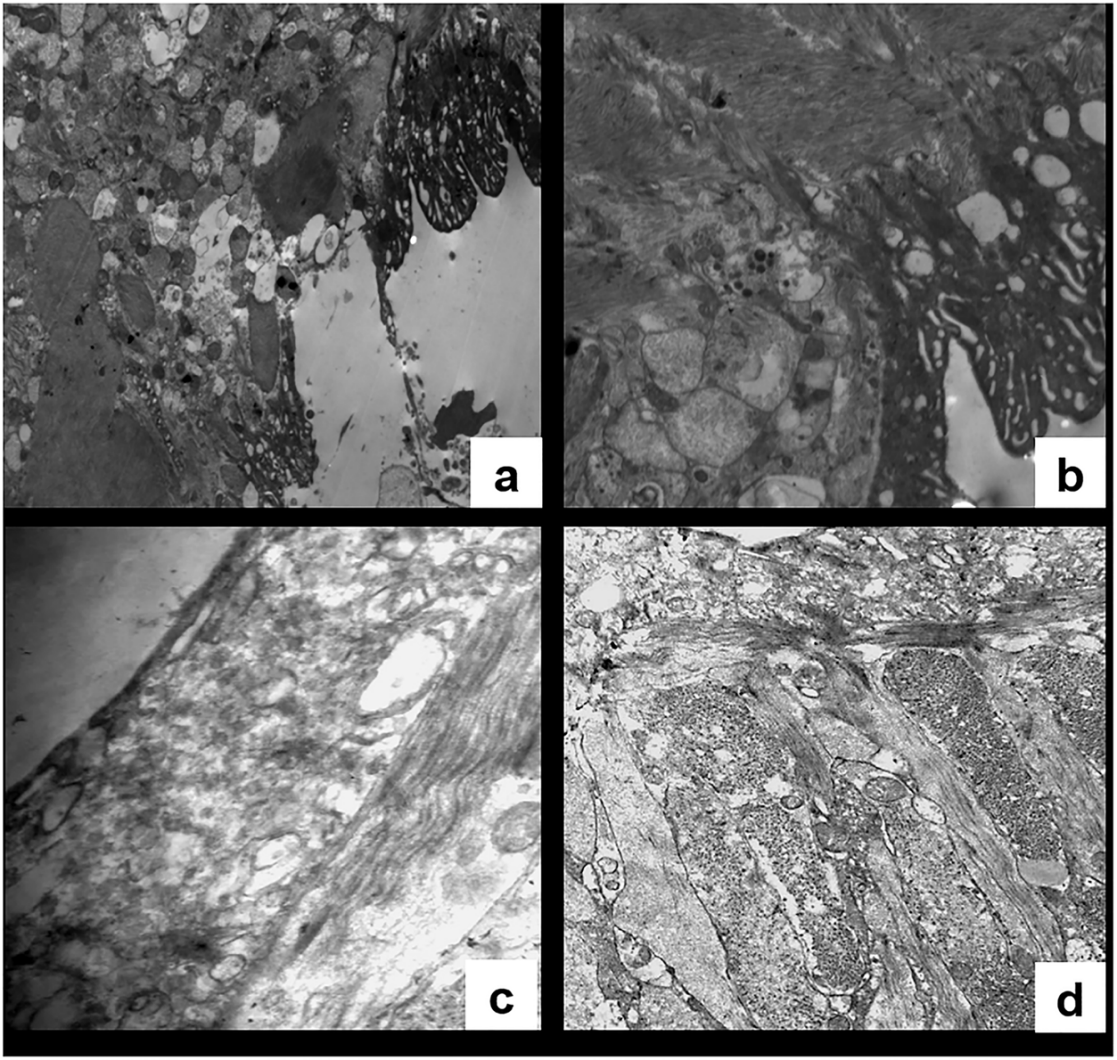
TEM of a male worm recovered from a mouse treated withMFS-LNC-OA showing. (a) Tegumental sloughing in certain areas (X2,000), (b) Marked tegumental irregularities and loss of tubercular spines with mesh like appearance (X4,000). (c and d) TEM of normal schistosoma worm included for comparison showing: (c) Dorsal tegumental surface (X7,500) and (d): Subtegumental tissue (X4,000).

TEM images of male worms recovered from mice treated with the same test formulation (MFS-LNC-OA) showed erosion of the tegument in some areas (Fig 7a) whereas, in others, it appeared intact with marked irregularities and loss of tubercle spines. Further, several vacuoles of variable size generated a mesh like appearance (Fig 7b). TEM images of a normal *S*. *mansoni* male worm (Fig 7c and 7d) are shown for comparison.

### Pharmacokinetic study

The LC-MS/MS method used for the quantification of MFS in plasma was linear within the concentration range 1–100 ng/mL(r^2^ ≥0.998). The limits for detection and quantification for MFS in plasma were less than 1 ng/ml. The coefficient of variation for intra-day and inter-day precision for three quality control MFS solutions (4, 20, 100 ng/mL) was 14 and 12% respectively. The MFS retention time was 3.4 min.

**Fig 8:**
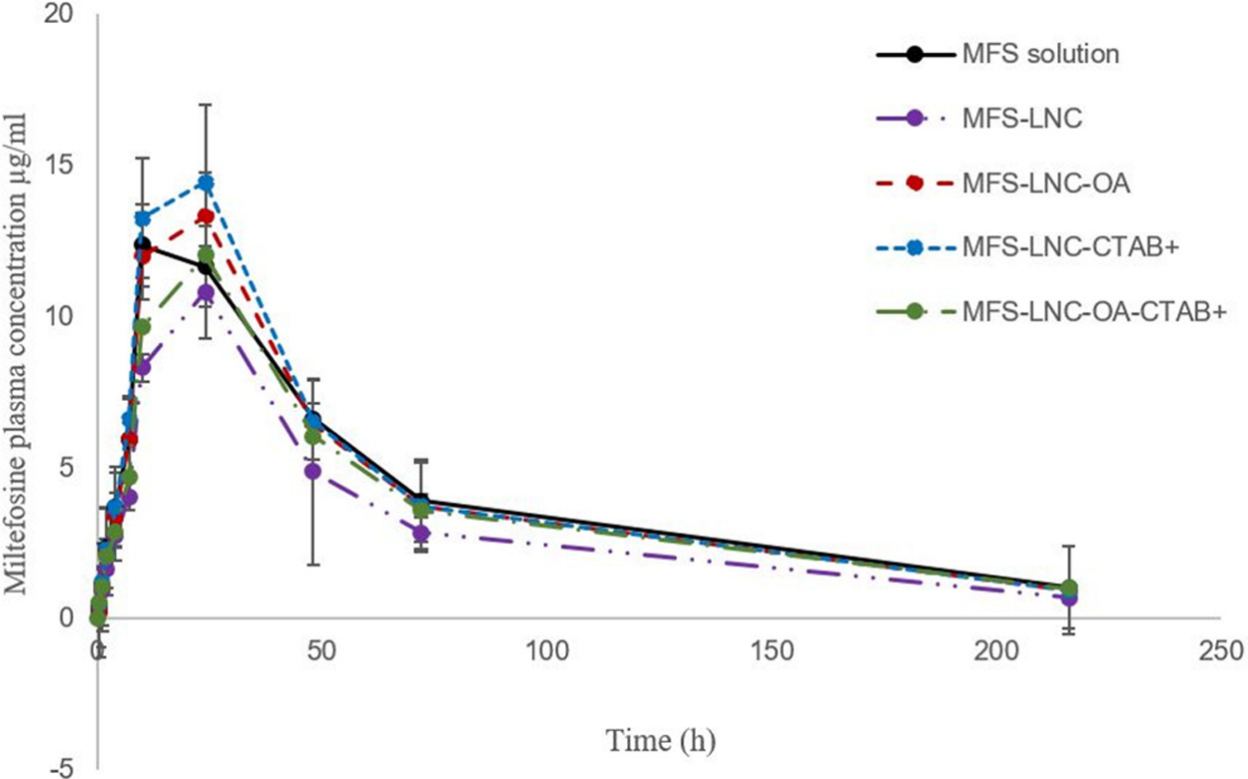
Mean plasma levels of miltefosine following oral administration of a 10 mg/kg dose of miltefosine as solution and lipid nanocapsule formulations to rats (n = 5). Error bars represent standard error of the mean.

Fig 8 shows the plasma concentration versus time profiles of MFS after oral administration of MFS solution and the test MFS-LNCs. The calculated PK parameters: maximum plasma concentration (C_max_), time to reach C_max_ (t_max_), half-life (t_½_) and mean residence time (MRT) are shown in Table 5. A significant increase in t_max_ values for all MFS-LNCs compared to MFS solution was observed (p <0.05). However, the differences between C_max_, t_½_, AUC and MRT for MFS solution and each of the test MFS-LNC formulations did not reach statistical significance.

**Table 5:** Pharmacokinetic parameters of miltefosine after a single oral dose (10 mg/kg) of MFs solution or MFS-LNCSs formulations in rats.

Parameter	MFS Solution	MFS-LNC	MFS-LNC-OA	MFS-LNC-CTAB^+^	MFS-LNC-OA-CTAB^+^
**C**_**max**_(μg/mL)	12.5±3.6	10.8±4.6	13.4±5.7	14.4±3.7	12.0±4.6
**t**_**max**_(h)	10±0	24±0^a^	24±0^a^	24±0^a^	24±0^a^
**t**_**1/2**_ (h)	62.6±6.5	63.6±1.4	64.9±10.4	64.1±5.5	70.2±6.1
**AUC**_**0-inf**_ (mg.h/L)	1012.8±59.8	769.7±99.3	1015.7±112.0	1069.9±208.6	957.2±164.3
**MRT** (h)	81.9±9.2	77.6±6.6	80.3±15.2	77.7±10.9	88.7±12.0

^a^p<0.05

## Discussion

In the treatment of schistosomiasis, oral administration provides for mass chemotherapy and patient compliance. Efficacy of oral treatment depends on drug bioavailability and interaction with therapeutic targets including mainly the fluke tegument, a prime site of vital and immunological functions [9, 12, 13]. Structurally, the tegument is bounded externally by a dual phospholipid anionic bilayer [12] which mediates affinity for some drugs and electrostatic interactions. LNCs, the nanocarrier selected to achieve the study objective were proven to enhance the intestinal permeability of some drugs [19, 29]. LNCs consist of a lipid Labrafac core and a thick pegylated tensioactive (Solutol)/phospholipid shell that provides for stealth properties in addition to storage and gastrointestinal stability [17, 18].

Incorporation of MFS, a membrane active phospholipid with intestinal barrier modulating effect [6, 30], reported to enhance drug efficacy [31, 32]into LNCs could generate bioactive nanovectors integrating MFS bioactivity and LNC characteristics, which can be further modulated by functional additives.

### Physicochemical characteristics of MFS-LNCS formulations

LNCs could be prepared using MFS as the only phospholipid component. Blank and MFS-LNCs with good colloidal properties (Table 2) were modified by incorporation of pharmaceutical functional additives. The smaller particle size of CTAB-containing LNCs can be attributed to a reduction of nanoemulsion globule size during preparation as MFS/CTAB mixture was reported to exhibit dense packing at the air/water interface with synergistic surface activity [33]. Lipoid was added to LNCs containing CTAB but not a mixture of CTAB and oleic acid to counterbalance the high CTAB hydrophilicity. Further, oleic acid, a long chain (C18) unsaturated fatty acid, probably fluidizing the surfactant monolayer [34]necessitated a lower heating/cooling temperature range (45–65°C) for the preparation of LNC-OA. Physical stability of different MFS-LNCs coupled with MFS retention upon storage at 4°C for 4 months suggested integrity of the MFS-LNCs structure.

TEM images revealed generally spherical and uniform LNCs except for MFS-LNC-OA which showed apparently composite vesicular structures (Fig 1a–1d). Unsaturated fatty acids are known to form vesicles and to rapidly incorporate into phospholipid bilayers [35, 36]. Although the TEM image of MFS-LNC-OA-CTAB^+^ did not show the oleic acid-induced vesicular structures, formation of more stable CTAB/oleic vesicles of the LNCs size range cannot be ruled out [37].

High EE% values (97.7 to 98.7; SD 0.36–0.98) despite water solubility and relatively small molecular size of MFS, implied integration of MFS within the stable LNC structure, most probably at the core/pegylated surfactant shell interface with the hydrophobic alkyl chain extending into the lipid core, a property of hexadecylphosphocholines[38]. This was verified by the high retention stability of MFS upon storage of LNCs at 4°C for 4 months. MFS as the phospholipid component of pegylated liposomes was proven to form a highly stable structure in buffer and plasma[39]. Fig 4 shows a hypothetical diagram of the MFS-LNCs under study. Nonetheless, further studies are needed to support the study findings.

Sustained release of MFS-LNCs at physiological pH for a 24 h study period with a limited burst effect (Fig 2) can be attributed to restricted destabilization of LNCs as a result of MFS embedding at the LNC core/shell interface, an observation reported previously for the amphiphilic drug amiodarone[40]. Slower release of MFS-LNC-CTAB^+^ compared to MFS-LNCs(p<0.05) can be explained by the dense MFS/CTAB interfacial packing [33]. Conversely, faster release from MFS-LNC-OA can be induced by the shell fluidizing effect of oleic acid and/or formation of vesicular assemblies with different release properties[34–36]. More studies are needed to understand the effect of fatty acids on LNCs properties. Despite differences with in vivo conditions, *in vitro* release data presumably indicated integrity of the MFS-LNCs structure during the 6 h gastrointestinal transit time in mice [41] with limited premature drug release.

Although MFS was administered orally, an *in vitro* hemolytic activity study was conducted to provide information on the effect of LNCs on MFS membrane activity and cytotoxicity.MFS is known to exert extensive hemolysis of erythrocytes, precluding intravenous administration [6, 42]. This was attributed to the formation of non-lamellar self-assembly structures that destabilize the erythrocyte membrane bilayer, an effect promoted by the cationic head group [43]. Nanocarriers were shown to suppress the lytic activity of some drugs including MFS [44, 45]. In this study, LNCs reduced MFS-induced hemolysis (Fig 3), MFS-LNC-OA-CTAB^+^ exerting 100% inhibition. Hemolysis data combined with high MFS entrapment efficiency (EE %) and slow release data (Fig 2) substantiated structural integrity of MFS-LNCs with preservation of the polar head-mediated MFS membrane activity. The relatively low hemolysis-suppressing effect of cationic MFS-LNC-CTAB^+^ can be attributed to the CTAB surface activity and dense interface packing of MFS/CTAB[30]. On the other hand, MFS-LNC-OA, with a much lower charge density and surface activity, exhibited a more pronounced hemolysis preventing effect. The notable hemolysis inhibition by MFS-LNC-OA-CTAB^+^ supports the formation of complex oleic acid/CTAB vesicular nanostructures or molecular interactions, reducing MFS membrane activity. Hemolysis data may have important implications in MFS bioactivity.

### Antischitosomal activity of MFS-LNC formulations

StandardMFS-LNCs (without additives), administered in a single 20mg/kg, significantly enhanced antischistosomal activity of MFS in *S*. *mansoni*-infected mice. Selection of a 20mg/kg was based on the daily oral dose of MFS in the treatment of leishmaniasis in mice[46]. Enhanced activity was manifested as significant reduction of adult worm load (Table 3) and hepatic granuloma size (Table 4) compared to the same dose of MFS solution and blank (drug-free) LNCS. Lack of antischistosomal activity of MFS solution coupled with poor in vitro MFS release from LNCs at physiological pH for 8 h (Fig 2) suggested implication of MFS-LNCs as native nanostructures combining MFS bioactivity and LNCs carrier properties in promoting intestinal transport and/or MFS-induced antischistosomal effects. In fact, MFS exerts fast killing of *S*. *mansoni* worms in vitro [47],crosses the intestinal epithelium via a non-specific passive pathway and opens epithelial tight junctions[6, 30]. Further, we reported earlier that MFS reduces the size of hepatic granuloma in *S*. *mansoni*-infected mice[8], an effect attributed to the drug accumulation in the liver[15, 48] possibly reducing the eggs ability to induce granulomatous responses and/or to immunomodulatory functions reported for MFS[49]. On the other hand, gastrointestinalstability of LNCs [18], vesicle-mediated trancytosis of drug transport[19, 50] and/or possible gut uptake and translocation as native nanocarriers[19] might explain the enhanced antischistosomal activity of MFS presented as LNCs.

Inclusion of CTAB and chitosan (CS) as positive charge imparting agents or oleic acid (OA) as membrane permeabilizer significantly enhanced the effect of MFS-LNCs on adult worm load (Table 3) and granuloma size(Table 4). Activity enhancement by both cationic agents suggest a positive effect on intestinal transport and/or tegument interaction of MFS-LNCs. Similarly, a cationic nanoemulsion enhanced the activity of a new antischistosomal drug by favoring drug uptake by the worm [51]. The greater membrane activity of MFS-LNC-CTAB^+^was in agreement with their hemolytic activity and pointed to preservation of structural integrity of MFS-LNC-CTAB^+^ post absorption. The role played by cationic properties was verified by the enhanced activity of another cationic formulation with post-inserted chitosan (MFS-LNC-CS^+^) and conversely, the reduced activity of anionic MFS-LNC-DCP^¯^. Chitosan nanoparticles were shown recently to cross the intestinal mucosa [52]. However, a lower charge density and the nature of post-inserted macromolecular chitosan coat may account for the lower chitosan-induced activity enhancement compared to CTAB.

MFS-LNCs containing the unsaturated fatty acid, oleic acid (OA), exerted the greatest antischistosomal effect expressed as reduction in worm load (Table 3) and granuloma size (Table 4),though worm load reduction was not statistically different from that of MFS-LNC-CTAB^+^. It is worth noting that the MFS-LNC-OA formulation was less hemolytic than MFS-LNC-CTAB^+^. Thus, this formulation was used for histopathological examination (Fig 5A). Mild histopathological changes coupled with granuloma size reduction wereboth indicative of liver protection. Oleic acid-induced activity enhancement may involve enhanced drug permeation as a result of membrane permeabilization and structural deformation of tight junctions [53] and/or promotion of the interaction with the tegument membrane due to fusogenic properties [36]. Indeed, fatty acids were reported to kill schistosomes by exposing antigens on tegumental surface to the host immune system, achieving significant improvement in clinical effects [54, 55]. Interestingly, combining CTAB and oleic acid in MFS-LNC-OA-CTAB^+^ significantly reduced antischistosomal activity (Tables 3 and 4) verifying total inhibition of hemolytic activity (Fig 3). Combined findings suggested suppression of MFS membrane activity as a result of molecular interactions or the formation of complex structures [56].

### Pharmacokinetic study

In order to gain more insight into the enhanced MFS antischistosomal activity achieved by LNCs, a pharmacokinetic study was carried out on four selected formulations in rats for possible pharmacodynamic/systemic exposure relationship. The MFS dose (10 mg/kg) was adjusted to account for species differences [26]. The time to reach maximal plasma concentration (t_max_) for MFS solution (10 h, Table 5) and the relatively long t_1/2_(62h, Table 5) were consistent with literature data obtained in rats, 4 to 48 h for t_max_ and 84 h for t_1/2_[57]. Lack of significant difference between the pharmacokinetic parameters C_max_, t_½_,AUC and MRT of each of the test formulations and MFS solution (Table 5) indicated similar plasma systemic exposure toMFS despite distinct differences in antischistosomal activity (Tables 3 and 4). The significant increase in t_max_ for MFS-LNC formulations can be explained by the relatively slow MFS release from LNCs (Fig 2) and possible maintenance of integrity of LNCs which need more timefor diffusion through the mucus barrier[58].

LNCs were demonstrated to enhance the pharmacological activity of orally administered drugs mainly by modifying pharmacokinetics. Enhanced bioavailability accounted for increased activity of various poorly absorbed drugs such as paclitaxel[59] and fondaparinux [29]. Moreover, LNCs were also reported to increase circulation time because of stealth properties conferred by the PEG chains at their surface[60], increasing the activity of paclitaxel [61]. Lack of change in MFS pharmacokinetic parameters by LNCs in the present study can be ascribed to the relatively high absolute bioavailability of MFS in rats 82%[57], its wide distribution to and accumulation in several internal organs, including the kidney, liver, lung, spleen and adrenal glands[62]and relatively long half-life[57].

Combined data of the preclinical and the pharmacokinetic studies tend to indicate that the antischistosomal activity enhancement of MFS by LNCs most probably involved the relatively stable MFS-LNCs and accordingly was not solely dependent on systemic exposure to molecular MFS. Such findings corroborated those of previous studies in pointing out that classical drug PK/PD relationships should be considered prudently for oral nanomedicines [62, 63]. In fact, the efficacy of paclitaxel against resistant tumors was shown recently to depend on the structural integrity of nanomedicines rather than the AUC post oral absorption [63], accounting for the lack of PD/PK correlations. Further, oral nanomedicines may increase efficacy by targeting the sites of pathology. This was evidenced in a recent study by selective enhancement of the bioavailability of oral amphotericin B nanoparticles to the lung, liver and spleen as target organs via gut uptake and translocation of stable nanoparticles [64].

In the present study, *in vitro* structural stability of MFS-LNCs, poor release characteristics and formulation-dependent antischistosomal activity suggested dependence of efficacy enhancement on the structural integrity of MFS-LNCs and their potential gut uptake and translocation to the target fluke tegument. Remarkably, this was verified by scanning electron microscopic visualization of nano-objects on the surface of the tegument of blood dwelling flukes in *Schistosoma mansoni*-infected mice (Fig 6d and 6e).

Data obtained in this study indicated that presentation of MFS as selected LNC oral formulations significantly enhanced its antischistosomal activity in mice, tentatively corroborating hemolytic activity. Modulation of MFS-tegument membrane interaction by specific effects of formulation additives implied preservation, at least in part, of native LNC structures. This may support translocation to the fluke surface and justify poor efficacy prediction by systemic exposure data. The study indicated that apart from increasing bioavailability of poorly absorbed drugs, nanocarriers may improve drug efficacy of well absorbed drugs via targeting therapeutic sites assuming structural integrity and gut uptake. Further pharmaceutical, parasitological and toxicological studies are underway to strengthen the study findings.

## Conclusions

Single oral dose alternative therapy of schistosomiasis mansoni is a challenging objective that might be achieved effectively using MFS, a drug with recently rediscovered antischistosomal activity, formulated as lipid nanocapsules (LNCs). MFS-LNCs integrating the membrane activity of MFS, the biopharmaceutical advantages of LNCs and the biological effects of CTAB and oleic acid offered unique attributes that significantly enhanced antischistosomal activity and liver protection in mice. Poor pharmacokinetic / pharmacodynamic relationship indicated that the efficacy of orally administered nanomedicines may not necessarily relate to systemic drug exposure, particularly for stable nanostructures incorporating drugs with good bioavailability that are taken up by the gut and translocated to a therapeutic target(s). Data obtained implied possible targeting via the oral route by selected nanocarriers. From a clinical standpoint, effective single oral dose therapy of schistosomiasis mansoni would promote repurposing of MFS as an antischistosomal drug for mass chemotherapy with lower gastrointestinal toxicity and put forward single dose oral MFS-LNCs as a potential alternative to praziquantel chemotherapy.
